# A novel, rapid, ultrasensitive diagnosis platform for detecting *Candida albicans* using restriction endonuclease‐mediated real-time loop-mediated isothermal amplification

**DOI:** 10.3389/fcimb.2024.1450199

**Published:** 2024-11-11

**Authors:** Yizhe Wang, Yuhong Zhou, Jingrun Lu, Honglan Yu, Yu Wang

**Affiliations:** Department of Clinical Laboratory, The First People’s Hospital of Guiyang, Guiyang, Guizhou, China

**Keywords:** *Candida albicans*, loop-mediated isothermal amplification, restriction endonuclease, real-time fluorescence detection, ERT-LAMP-CA

## Abstract

**Introduction:**

*Candida albicans* (*C. albicans*, CA) is an essential invasive fungus in clinical diagnosis. Although several detection methods exist, none meet the need for early diagnosis. A rapid, sensitive, and specific diagnostic tool is crucial for effective prevention and control of *C. albicans* infections.

**Methods:**

This study aimed to develop a new, rapid, and ultrasensitive diagnostic tool for *C. albicans* detection based on restriction endonuclease-mediated real-time loop-mediated isothermal amplification (ERT-LAMP-CA). The ERT-LAMP-CA technology combines LAMP amplification, restriction endonuclease cleavage, and real-time fluorescence detection in a single reaction tube, which can complete a diagnosis of *C. albicans* in a short time (approximately 1 h).

**Results:**

Herein, we developed the primer sequences required for ERT-LAMP-CA based on the ITS2 gene of *C. albicans* and found that ERT-LAMP-CA limit of detection was approximately 500 ag/μL genomic DNA and can present negative results for non-*C. albicans* templates. We tested sputum samples from 64 patients with suspected *C. albicans* infections to validate ERT-LAMP-CA applicability in clinical sample testing and found that ERT-LAMP-CA was consistent with multiplex PCR-capillary electrophoresis.

**Discussion:**

In conclusion, ERT-LAMP-CA is a rapid, accurate, and sensitive assay with excellent potential for clinical and basic laboratory diagnosis and an efficient screening strategy.

## Introduction

Invasive fungal diseases have emerged as one of the leading causes of human disease since the early 1980s (Brown et al., 2012). Globally, > 150 million severe fungal infections occur annually, resulting in approximately 1.7 million deaths (Kainz et al., 2020). Severe invasive fungal diseases and deaths are more common in immunocompromised populations and those with severe underlying diseases (Wilson et al., 2002). These patients suffer from time-consuming diagnoses, extended treatment periods, expensive treatments, and poor treatment outcomes (Benedict et al., 2019). Simultaneously, recent studies have reported that drug-resistant fungi are becoming increasingly common (Vitiello et al., 2023). Cross-resistance between azoles and echinocandins has been reported in drug-resistant yeasts, including *Candida albicans* (*C. albicans*), which has a significant impact on clinical diagnosis (Arendrup and Patterson, 2017).

*C. albicans*, or *Pseudo hyphae albicans*, is a common cause of invasive fungal disease. The World Health Organisation classified *C. albicans* as a critical priority group, one of the four pathogenic fungi of most significant concern (Casalini et al., 2024). *C. albicans* is among the most common co-infecting fungi, especially in COVID-19 patients (Chen et al., 2020). *C. albicans* is a commensal yeast fungus on the oral, gastrointestinal, and genital mucosal surfaces and skin of humans. It has the potential to become an opportunistic pathogen that can cause mucosal-associated diseases and even life-threatening systemic infections when antibiotic-induced dysbiosis, artificial immunosuppression, and compromised mucosal barrier integrity occur (Pappas et al., 2018; Lopes and Lionakis, 2022). Some patients miss the optimal time for treatment due to a lack of early definitive diagnosis, resulting in an increased mortality rate (Adams et al., 2018; Proctor et al., 2023). Therefore, rapid and accurate *C. albicans* detection is essential for early prevention, control, and treatment of invasive candidiasis. Simultaneously, early treatment prevents the emergence of drug-resistant strains.

Detecting *C. albicans* in the clinic primarily relies on the traditional culture method. Although the culture of pathogens is the gold standard for diagnosing the pathogenicity of fungal infections, it is time-consuming, insensitive, and does not meet the clinical therapeutic requirements (Pincus et al., 2007; Wickes and Romanelli, 2020). Immunofluorescence methods and mass spectrometry were employed to overcome the time-consuming problem of the “gold-standard” culture method (Zehm et al., 2012; Gunasekera et al., 2015). Although these two methods of *C. albicans* detection can shorten the clinical testing time, they must be operated and analysed by professionally trained personnel, which is challenging to popularise because of the high demands on practitioners and laboratory instruments. The traditional fluorescence quantitative PCR amplification has become a common method for fungi identification. However, this method is time-consuming and usually results in non-specific amplification, leading to misclassification or missed results (Kasai et al., 2006; Ramos et al., 2017).

Researchers have developed several isothermal amplification methods for nucleic analysis, including loop-mediated isothermal amplification (LAMP), multiplexed cross-substitution amplification, and recombinant enzyme polymerase amplification to address the shortcomings of the current clinical detection methods. These methods do not require thermal cycling instruments. They can identify infections in real time, which is ideal for medical units at all levels. Notomi, a Japanese scholar, described LAMP, a new gene diagnostic technology in Nucleic Acids Res (Notomi et al., 2000). The LAMP involves designing four kinds of specific primers for six regions of the target gene and amplifying the gene template, primers, and strand-substituted DNA synthetase at a constant temperature of 60–65°C under the action of Bst DNA polymerase (Nagamine et al., 2002). Bst DNA polymerase amplifies the gene template, primers, and strand-substitution DNA synthetase at a constant temperature of 60–65°C, resulting in a 10^9^–10^10^-fold nucleic acid amplification in approximately 15–60 min (Parida et al., 2008). The LAMP technique considerably reduces analysis time, which has increased its applicability in pathogen diagnostics (Dhama et al., 2014). Because of its high sensitivity, it can identify pathogens in small samples. Studies at this stage include the detection of COVID-19 (Choi et al., 2023), hepatitis B (Jayanath et al., 2018), parasites (Mugambi et al., 2015), and others using LAMP. The laboratory has previously published studies on *C. albicans* detection using the LAMP-LFB technique (Wang et al., 2022). However, the LFB method involves the problems of open caps and the inability to quantify, which were solved in this study based on laboratory studies.

This study used the LAMP technique and real-time fluorescence analysis to diagnose *C. albicans* infection. This method can produce faster and more accurate results than traditional culture and fluorescence quantitative PCR methods because the previous coronavirus detection has made PCR detection instruments and professionals more widely available than immunofluorescence and mass spectrometry (Luchi et al., 2023). It is less likely to contaminate amplification products than isothermal amplification-fluorescence chromatography. It can produce more accurate quantitative test results (Khunger et al., 2024).

## Materials and methods

### Reagents and apparatus

The grinding reagents for nucleic acid extraction and purification of clinical samples and the multiplex PCR-capillary electrophoresis reagents for detecting lower respiratory tract fungi were obtained from Shanghai Meiji Yuhua Biomedical Technology Co. (China). Ex-DNA bacterial genome/Ex-DNA virus genome nucleic acid extraction and purification kits were purchased from Xi’an Tianlong Science and Technology Co. (China). An isothermal amplification kit (RNA/DNA universal), a polymer nanoparticle-based lateral flow biosensor (LFB), and a visual detection reagent were obtained from Tianjin Huidexin Science and Technology Development Co, Ltd. (China). *BsrDI* enzyme and buffer were obtained from New England Biolabs (USA). The nucleic acid extraction and purification instrument GeneRotex96 was obtained from Xi’an Tianlong Technology Co., Ltd; the real-time fluorescence amplifier ABI7500 was obtained from ABI(USA); the nucleic acid electrophoresis instrument JY300E was obtained from Beijing Junyi Company(China); and the gel imaging system E-BOX was obtained from Vilber Bio-Imaging Company, Ltd(France). The concentrations of the samples used in the experiments were detected using a NanoDrop ONE A260/A280 spectrophotometer from Thermo Fisher Scientific(USA).

### Fungi, bacterial strains, viruses and clinical samples

**Table 1** shows the 32 strains of pathogen used in this study, including two strains of *C. albicans* and 30 strains of non-*C. albicans*. The reference strains of *C. albicans* were obtained from the American Type Culture Collection (ATCC 10231). Other strains were isolated from clinical specimens and identified using the gold-standard method of the Department of Medical Laboratory of *the First People’s Hospital of Guiyang*. All strains were isolated in 15% (w/v) glycerol broth and stored at –80°C. DNA of *C. albicans* was used as a positive control, primarily to analyse the assay performance and determine the optimal reaction temperature. *Candida glabrata* and *Pseudomonas aeruginosa* DNA were used as negative controls.

**Table 1:** Pathogens used in this study.

Pathogens	Strain no. (source of strains)^a^	No.of strains	RT-LAMP-CA assay^b^
*Candida albicans*	ATCC 10231	1	P
*Candida albicans*	Isolated strains (GFPH)	1	P
*Klebsiella Pneumoniae*	Isolated strains (GFPH)	1	N
*Stenotrophomonas maltophilia*	Isolated strains (GFPH)	1	N
*Streptococcus agalactiae*	Isolated strains (GFPH)	1	N
*Acinetobacter baumannii*	Isolated strains (GFPH)	1	N
*Enterococcusfaecalis*	ATCC 29212	1	N
*Bacteroidesfragilis*	Isolated strains (GFPH)	1	N
*Bordetella pertussis*	Isolated strains (GFPH)	1	N
*Pseudomonas aeruginosa*	ATCC 27853	1	N
*Streptococcus pneumoniae*	ATCC 49619	1	N
*Proteusbacillus vulgaris*	Isolated strains (GFPH)	1	N
*Haemophilus influenzae*	ATCC 10211	1	N
*Staphylococcus epidermidis*	Isolated strains (GFPH)	1	N
*Staphylococcus aureus*	ATCC 29213	1	N
*Crytococcus Neoformans*	Isolated strains (GFPH)	1	N
*Serratia marcescens*	Isolated strains (GFPH)	1	N
*Chlamydia pneumonia*	Isolated strains (GFPH)	1	N
*Enterobacter cloacae*	Isolated strains (GFPH)	1	N
*Escherichia coli*	ATCC 25922	1	N
*Aspergillusflavus*	Isolated strains (GFPH)	1	N
*Candida glabrata*	Isolated strains (GFPH)	1	N
*Candida tropicalis*	Isolated strains (GFPH)	1	N
*Mycoplasma pneumoniae*	Isolated strains (GFPH)	1	N
*Adenovirus*, Adv	Nucleic acid retention of clinical specimens (GFPH)	1	N
*Corona Virus Disease 2019*, COVID-19	Nucleic acid retention of clinical specimens (GFPH)	1	N
*Influenza A virus*, FluA	Nucleic acid retention of clinical specimens (GFPH)	1	N
*Influenza B virus*, FluB	Nucleic acid retention of clinical specimens (GFPH)	1	N
*Respiratory syncytial virus*, RSV	Nucleic acid retention of clinical specimens (GFPH)	1	N
*Human parainfluenza virus*, HPIV	Nucleic acid retention of clinical specimens (GFPH)	1	N
*Human metapneumovirus*, HMPV	Nucleic acid retention of clinical specimens (GFPH)	1	N
*Rhinovirus*, RhV	Nucleic acid retention of clinical specimens (GFPH)	1	N

^a^GFPH, Guiyang First People's Hospital; ATCC, American Type Culture Collection.

^b^P, positive; N, negative. Only the genomic DNA template of C. albicans could be detected by the ERT-LAMP-CA detection system, indicating that the method has a very high specificity.

We collected 64 human sputum samples between August 2023 and March 2024 in the Laboratory of *the First People’s Hospital of Guiyang* to examine the applicability of the ERT-LAMP-CA assay in clinical samples. We identified 18 samples as *C. albicans*-positive using the gold standard method. All collected samples were stored at –80°C before use.

### Response mechanism of *C. albicans*-LAMP-LFB and ERT-LAMP-CA assays

**Figure 1:**
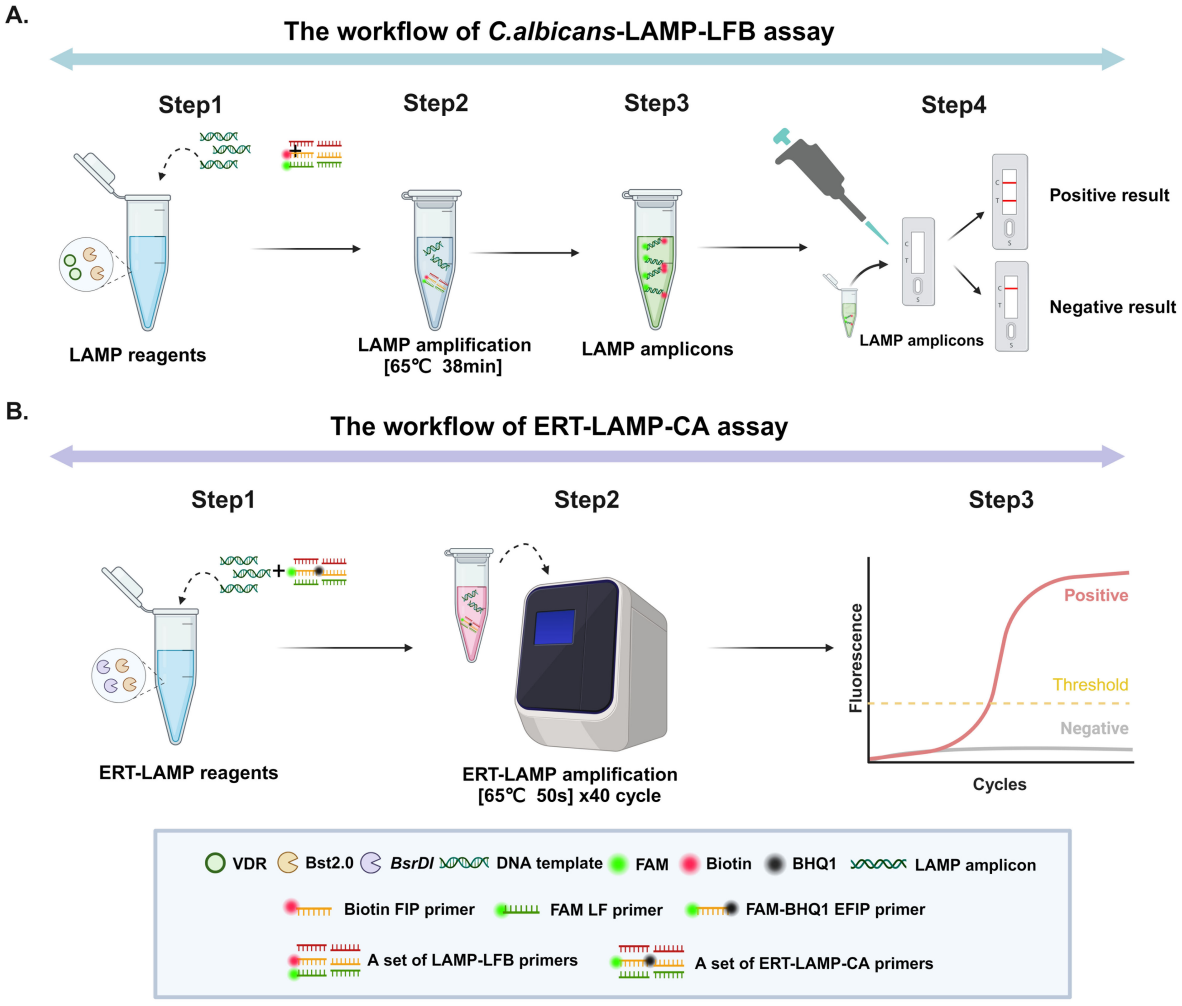
Operation of the *C.albicans* detection system. Detailed description of the *C*. *albicans* LAMP-LFB assay process. The whole detection process can be summarised in four main steps: the first step is the construction of the reaction system, the second step is the LAMP amplification process, the third step is the use of the naked eye to observe changes in specific stains for analysis, and the fourth step is the detailed analysis of the results by LFB. **(A)** Detailed description of the ERT-LAMP-CA assay process. The whole detection process is mainly divided into three steps: the first step is the construction of the ERT-LAMP-CA amplification system, the second step is the amplification of the ERT-MCDA-CA detection system on a real-time fluorescence amplifier, and the third step is the analysis and judgement of the results according to the changes in real-time fluorescence absorption **(B)**.

**Figure 1** displays the reaction mechanisms of *C. albicans*-LAMP-LFB and ERT-LAMP-CA assays. In the *C. albicans*-LAMP-LFB assay, an effective diagnosis of *C. albicans* was achieved using a combination of the LAMP reaction and the LFB detection technique. This method employed a FAM-labelled ring primer for the LAMP reaction, followed by detecting the reaction product using the LFB detection technique. A red line in the TL region of the LFB indicates a positive test result in the ERT-LAMP-CA assay; however, the reaction mechanism combines the LAMP reaction with real-time fluorescence detection technology. This system introduced an EFIP primer with fluorescent and quenching groups, and a real-time fluorescence quantitative PCR instrument monitored the fluorescence signals during amplification. A fluorescence amplification curve indicates a positive test result.

### Design of ERT-LAMP-CA primers and standard plasmid construction

**Figure 2:**
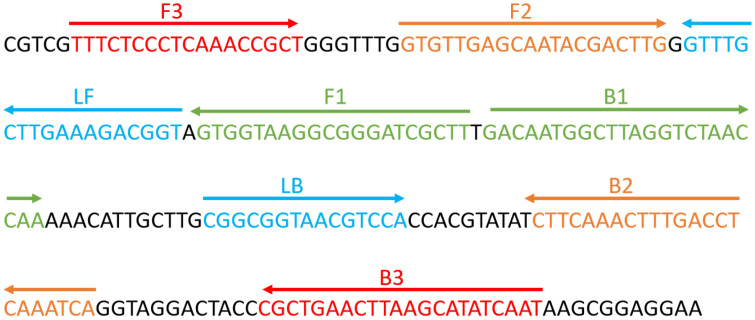
Sequence and position of the ITS2 gene used to design loop-mediated isothermal amplification primers. The nucleotide sequence of the ITS2 sense strand is presented. Right and left arrows indicate the sense and complementary sequences used, respectively.

The primers required for the LAMP method include six different regions of a specific sequence. Herein, based on the ITS region of the rRNA gene of *C. albicans*, primers were designed using primer software PREMIER 5.0 Primer Explorer version 4 (Eiken Chemical) according to previous research (Wang et al., 2022). For real-time fluorescence measurement, FAM (6-carboxyfluorescein) was labelled at the 5’ end of the FIP primer, while BHQ1 was used as a quencher and labelled at the 3’ end. **Figure 2** and **Table 2** show the positions and sequences of LAMP primers. Primer synthesis was performed by Beijing Qingke Biotechnology Co., Ltd. (China).

**Table 2:** Primers used in this study.

RT-LAMP-C.A Assay Primers			
Primers name	Sequences and modifications (5’-3’) ^a^	Length^b^	Gene
Ca-F3	TTTCTCCCTCAAACCGCT	18nt	ITS2
Ca-B3	ATTGATATGCTTAAGTTCAGCG	22nt	
Ca-FIP	AAGCGATCCCGCCTTACCAC-GTGTTGAGCAATACGACTTG	40mer	
Ca-EFIP*^c^	5’-FAM-TGCAATG-AAGCGAT-(BHQ1)-CCCGCCTTACCAC-GTGTTGAGCAATACGACTTG	48mer	
Ca-BIP	GACAATGGCTTAGGTCTAACCAA-TGATTTGAGGTCAAAGTTTGAAG	46mer	
Ca-LF	ACCGTCTTTCAAGCAAAC	18nt	
Ca-LB	CGGCGGTAACGTCCA	15nt	

^a^FAM, 6-carboxy-fluorescein; BHQ1, Fluorescence quenching group.

^b^Mer, monomeric; nt, nucleotide.

^c^Ca-EFIP^*^, Restrictionendonuclease recognition sequence, FAM and BHQ1 labeled.

### The ERT-LAMP-CA assay for *C. albicans* detection

The reaction system (25 μL) for the LAMP assay consisted (Wang et al., 2022) of 12.5 μL of 2 × isothermal reaction buffer, 0.4 μm of each outer primer, F3 and B3, 0.8 μm of each loop primer, LF and LB, 1.6 μm of each inner primer, EFIP, and BIP. Bst 2.0 DNA polymerase (8 U) 1.0 μL, *BsrDI* 1.0 μL, NEBuffer™ r2.1 2.5 μL and pure medium template 2 μL (5 μL for clinical samples). The reaction was performed for 40 cycles using the real-time fluorescence instrument, setting the mode to 65°C for 50 s. Negative control mixtures contained *Candida glabrata* and *Pseudomonas aeruginosa* genomic DNA, and blanks were prepared using 2 µL double-distilled water. The ERT-LAMP-CA real-time fluorescence detector and 1.5% agarose gel electrophoresis validated the assay.

### Optimization of reaction temperature for the ERT-LAMP-CA assay

The optimal reaction temperature for the ERT-LAMP-CA assay was confirmed using the ITS2 plasmid. Amplification products were monitored using a real-time fluorescence quantitative PCR instrument (ABI 7500) at reaction temperatures from 60–67°C (1°C interval). An S-shaped amplification curve indicated specific amplification.

### Detection sensitivity of the ERT-LAMP-CA assay

Serial dilutions of the plasmids were amplified to confirm the limit of detection (LoD) of the ERT-LAMP-CA assay. The LoD of the ERT-LAMP-CA assay was defined as the lowest serial dilution of the standard plasmid that could be detected in ≥ 95% of the tests performed at the study concentration. The *Candida albicans* nucleic acid template at an initial concentration of 10 ng/μL was gradient diluted using sterilised water to 1 ng/μL, 10 pg/μL, 1 pg/μL, 500 fg/μL, 50 fg/μL, 5 fg/μL, and 100 ag/μL. The *Candida albicans* nucleic acid template at the second concentration dilution was utilised to dilute to 1 pg/μL, and the same Gradient dilutions to 500 fg/μL, 50 fg/μL, 10 fg/μL, 1 fg/μL, and 500 ag/μL were performed using sterilised water.

### Specificity of the ERT-LAMP-CA assay

The nucleic acid of 32 strains was tested according to the optimal amplification temperature and time to assess the specificity of the ERT-LAMP-CA assay system in **Table 1**. A real-time fluorescence detector and 1.5% agarose gel electrophoresis were used to validate the results.

### Applicability of the ERT-LAMP-CA assay in clinical specimens

We collected 64 sputum samples from patients with suspected *C. albicans* infection in *the First People’s Hospital of Guiyang* to evaluate the ERT-LAMP-CA assay feasibility in clinical samples. The multiplex PCR-capillary electrophoresis (MPCR-CE) kit was from Chongqing Pasteur Biotechnology Co., Ltd. (China).

### Statistical analysis

A schematic diagram of the experimental process was created using BioRender.com. The data analysis was performed using IBM Statistical Package for the Social Sciences software (version 26), GraphPad Prism software, R language (version 3.6.3), and the Hiplot Platform.com.

## Results

### Validity of primer sets for ERT-LAMP-CA assay

**Figure 3:**
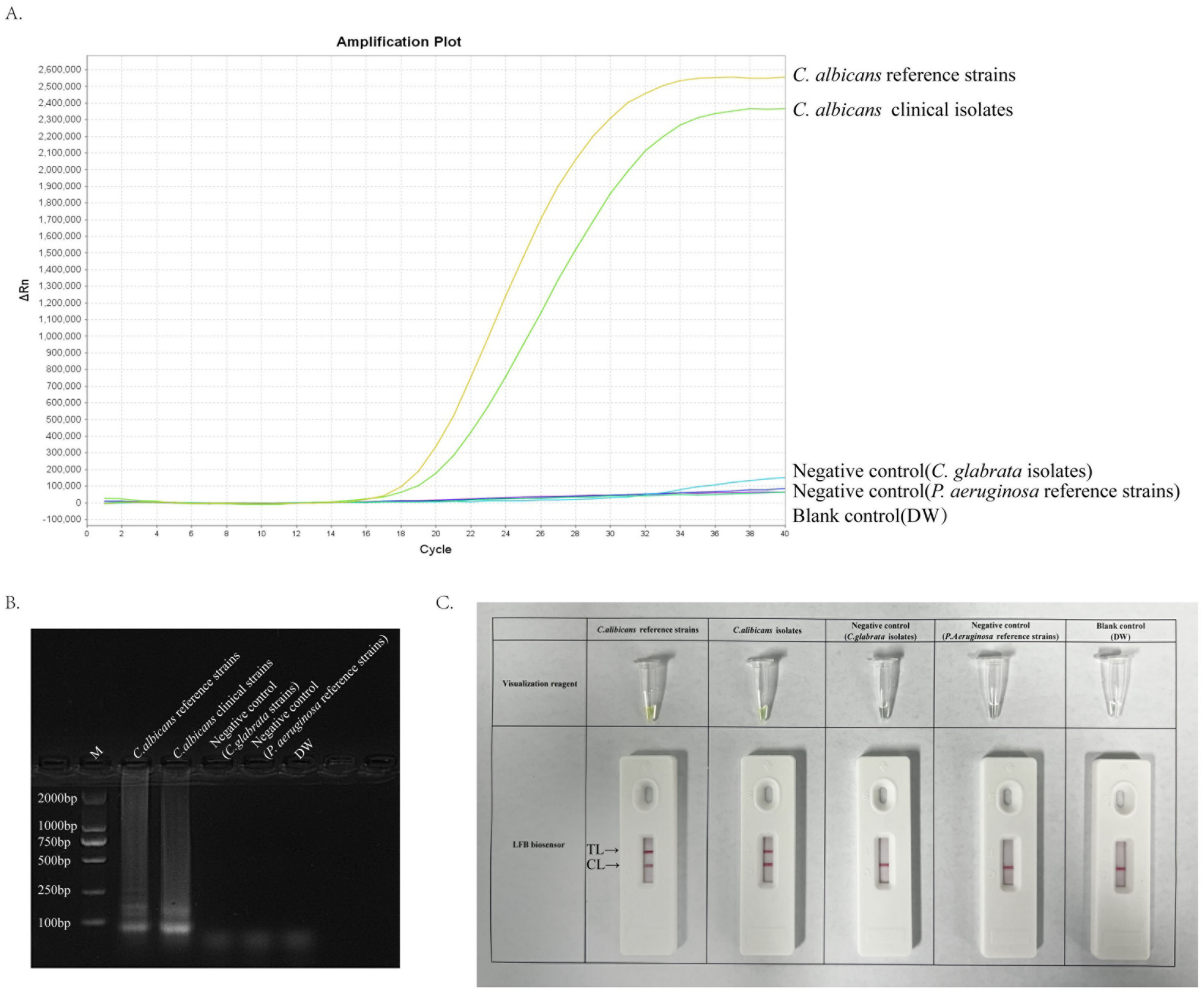
Validity of primer sets for ERT-LAMP-CA assay. Viability verification of ERT-LAMP-CA reaction by fluorescently labelled primer sets. *C.albicans* reference strains and clinical isolates were efficiently amplified in the ERT-LAMP-CA reaction at 64°C. Negative controls (Candida glabrata isolates and Pseudomonas aeruginosa reference strains), and blank controls (DW) did not respond. LAMP products were analysed by two assays: real-time fluorescence quantification **(A)**. Agarose gel electrophoresis **(B)**. Visualization reagent and LFB biosensor **(C)**.

A 40-minute LAMP reaction at 64°C was performed using DNA templates extracted from the standard strain of *C. albicans* (ATCC 10231) to verify the validity of the primer set for the ITS2 gene of *C. albicans*. The entire response was monitored using a real-time fluorescence PCR instrument for real-time fluorescence monitoring, and the final results were analysed using fluorescence detection and agarose gel electrophoresis. Furthermore, primer sets with specific fluorescent groups were used and detected using the difference in dye colour development and LFB after the reaction. **Figure 3** depicts that the *C. albicans* template was efficiently amplified, whereas the negative control strain template and Distilled water(DW)did not show any reaction. Accordingly, we confirmed that this primer set was suitable for establishing an ERT-LAMP-CA assay.

### The optimal temperature for ERT-LAMP-CA assay

**Figure 4:**
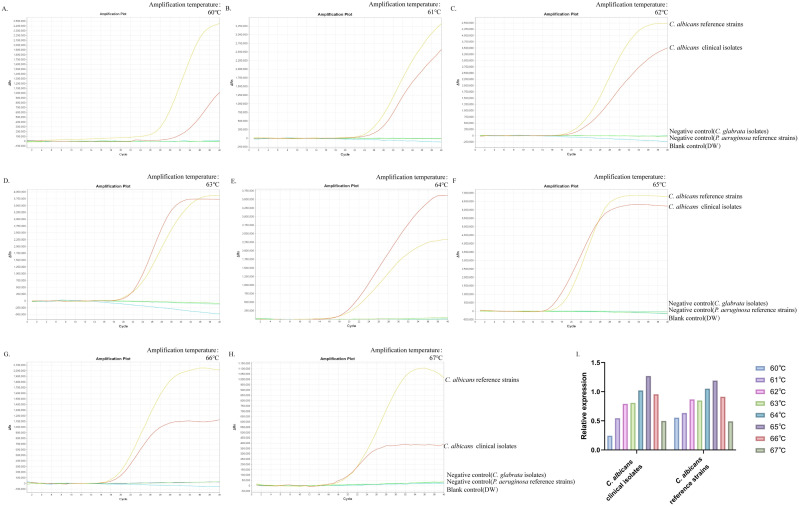
Optimal temperature for ERT-LAMP-CA assay. Temperature optimisation of ERT-LAMP-CA assay.A temperature gradient (60-68°C) was used to analyse the fluorescence acquisition of ERT-LAMP-CA using a real-time fluorescence PCR analysis system **(A-H)**. A relative quantitative comparison of the fluorescence amplification curves obtained from the *C.albicans* standard strain and the clinical strain at different temperatures from 60 to 68°C was carried out using a CT value of 40 labelled as 0 and the CT value of the *C.albicans* standard strain in the feasibility analysis labelled as 1. It can be concluded that 65°C is the optimal temperature for the amplification reaction **(I)**.

This study aimed to determine the optimum temperature for the ERT-LAMP-CA assay to optimise the reaction system. This study set up a temperature gradient from 60–67°C, with a 1°C interval between each temperature point, and used standard strain DNA of *C. albicans*, clinical isolate DNA, and negative controls (*Candida glabrata* isolated strain DNA, *Pseudomonas aeruginosa* standard strain DNA, and DW) in each reaction. A real-time fluorescence PCR instrument was utilised to perform the real-time fluorescence-LAMP amplification process, and the corresponding fluorescence data were collected and analysed. Eight fluorescence analysis plots corresponding to different temperatures were obtained (**Figures 4A–H**).

The results showed that the *C. albicans* ITS2 gene was successfully amplified at all temperatures. The results of the validity experiments were used as a benchmark, and the final cycle number was set to 40 cycles, 0 for a CT(Cycle threshold) value of 40 and 1 for the CT value of DNA amplification of the standard strains, to perform a simple analysis of the amplification efficiency at different temperatures (**Figure 4I**). The study showed that the fluorescence threshold was first reached at 65°C and exhibited the highest amplification efficiency. Therefore, it could be determined that 65°C was the optimal temperature for this reaction system.

### Sensitivity of the ERT-LAMP-CA assay

**Figure 5:**
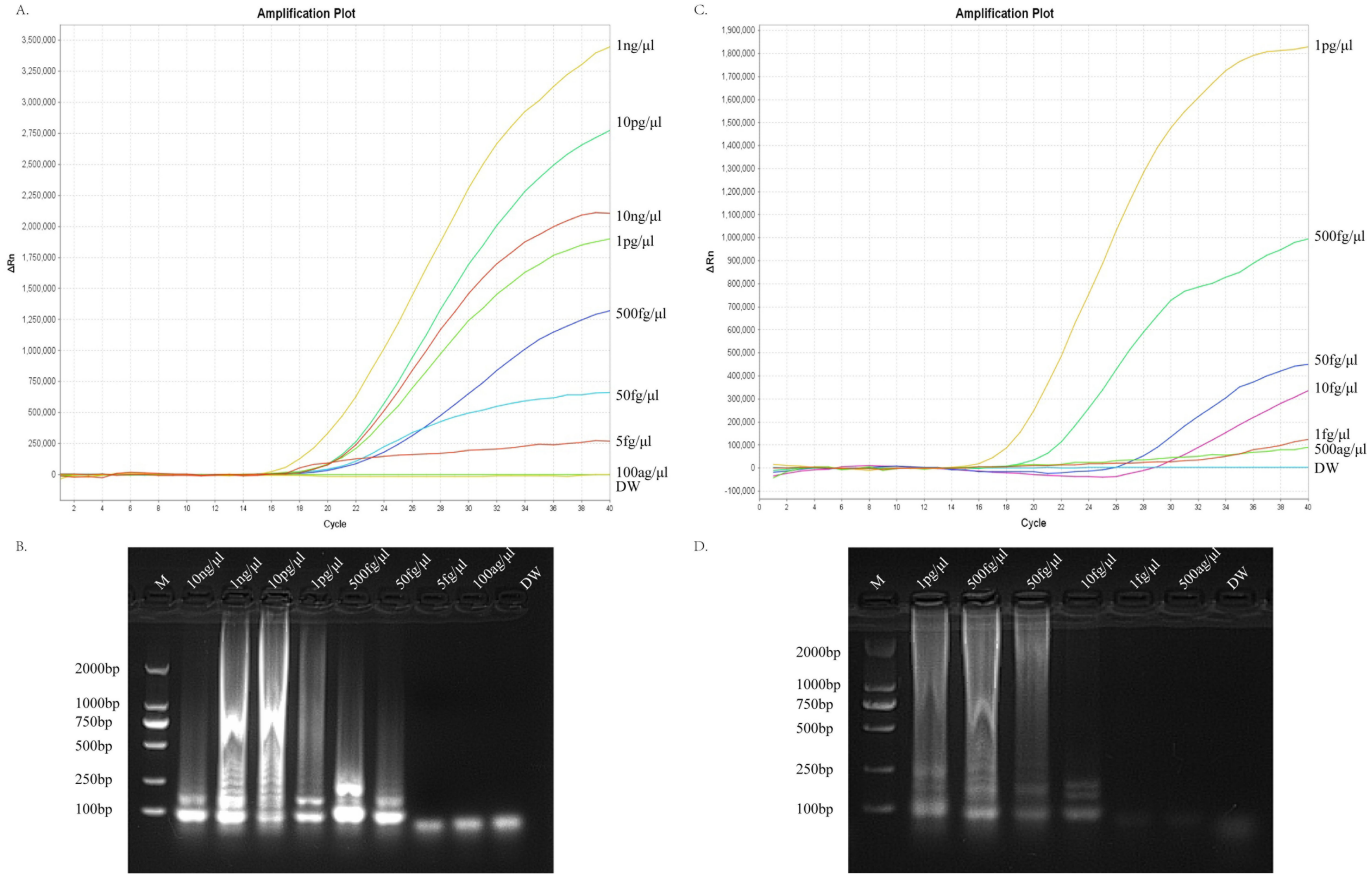
Sensitivity of the ERT-LAMP-CA assay.Sensitivity analysis of ERT-LAMP-CA assay. Genomic DNA was diluted sequentially:10ng/μl, 1ng/μl,10pg/μl, 1pg/μl,500fg/μl, 50fg/μl, 5fg/μl, 100ag/μl, blank control (DW) **(A, B)**. Genomic DNA was diluted sequentially:1pg/μl, 500fg/μl,50fg/μl, 10fg/μl,1fg/μl, 500ag/μl, blank control (DW) **(C, D)**. LAMP products were analysed using two assays: real-time fluorescence quantification and agarose gel electrophoresis.

The genomic DNA of a standard strain of *C. albicans* was used in this study and diluted according to a decreasing concentration gradient (10 and 1 ng/μL, 10 and 1 pg/μL, 500, 50, and 5 fg/μL, and 100 ag/μL) as a template for evaluating the sensitivity of the RT-LAMP assay system to assess the ERT-LAMP-CA assay sensitivity. **Figures 5A, B** demonstrate the monitoring of the amplification process using a real-time fluorescence PCR instrument and recording the corresponding fluorescence data for analysis. Furthermore, the amplification products were analysed using agarose gel electrophoresis, and the results showed that the lowest detection limit of the assay system was between 5 fg/μL and 100 ag/μL.

New concentration gradient dilutions (1 pg/μL, 500, 50, 10, and 1 fg/μL, and 500 ag/μL) were performed in this study and analysed using the same method to further validate the detection sensitivity. **Figures 5C, D** show that the lowest detection limit was approximately 500 ag/μL. This result is similar to the lower detection limit of the *C. albicans* LAMP-LFB assay previously studied by our experimental group.

Additionally, a negative result (CT value of 40) was defined as 0, and a CT value of 1 pg/μL was defined as 1 in this study, which was analysed based on the relative quantification of CT values. Based on the results of the two concentration gradient experiments, we performed a linear analysis of the DNA concentration in the reaction (**Supplementary Figure S1**), which revealed that the ERT-LAMP-CA assay system presented better linearity in the low concentration range.

### Specificity of the ERT-LAMP-CA assay

**Figure 6:**
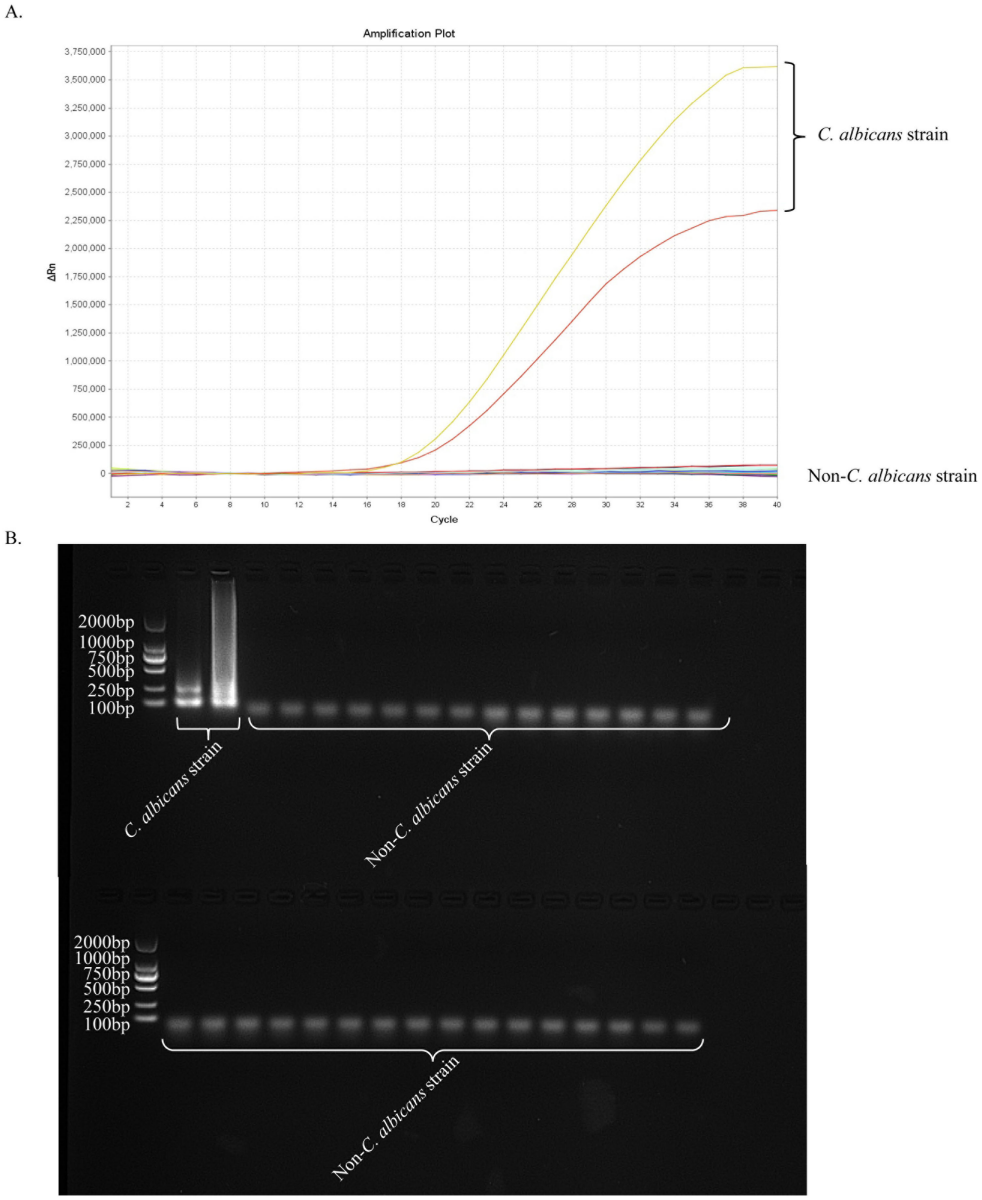
Specificity of the ERT-LAMP-CA assay.Confirmation of the specificity of the ERT-LAMP-CA detection system. The ERT-LAMP-CA assay system tested nucleic acid templates of 2 C.albicans standard strain and 30 non-C.albicans strains. None of the non-*C.albicans* strains showed amplification reactions. LAMP products were analysed by two assays: real-time fluorescence quantification **(A)** and 1.5% agarose gel electrophoresis **(B)**.

Nucleic acid templates of *C. albicans* strains and non-*C. albicans* strains or viruses were analysed using real-time fluorescence detection in this study to assess the specificity of the ERT-LAMP-CA assay (**Table 1**). **Figure 6A** illustrates that only the DNA templates of *C. albicans* strains showed fluorescence amplification (positive results). However, all non-*C. albicans* strains showed amplification-negative results in the ERT-LAMP-CA assay. We subjected the amplification products to agarose gel electrophoresis to obtain the same results (**Figure 6B**).

### Validation of the ERT-LAMP-CA assay in clinical specimens

**Figure 7:**
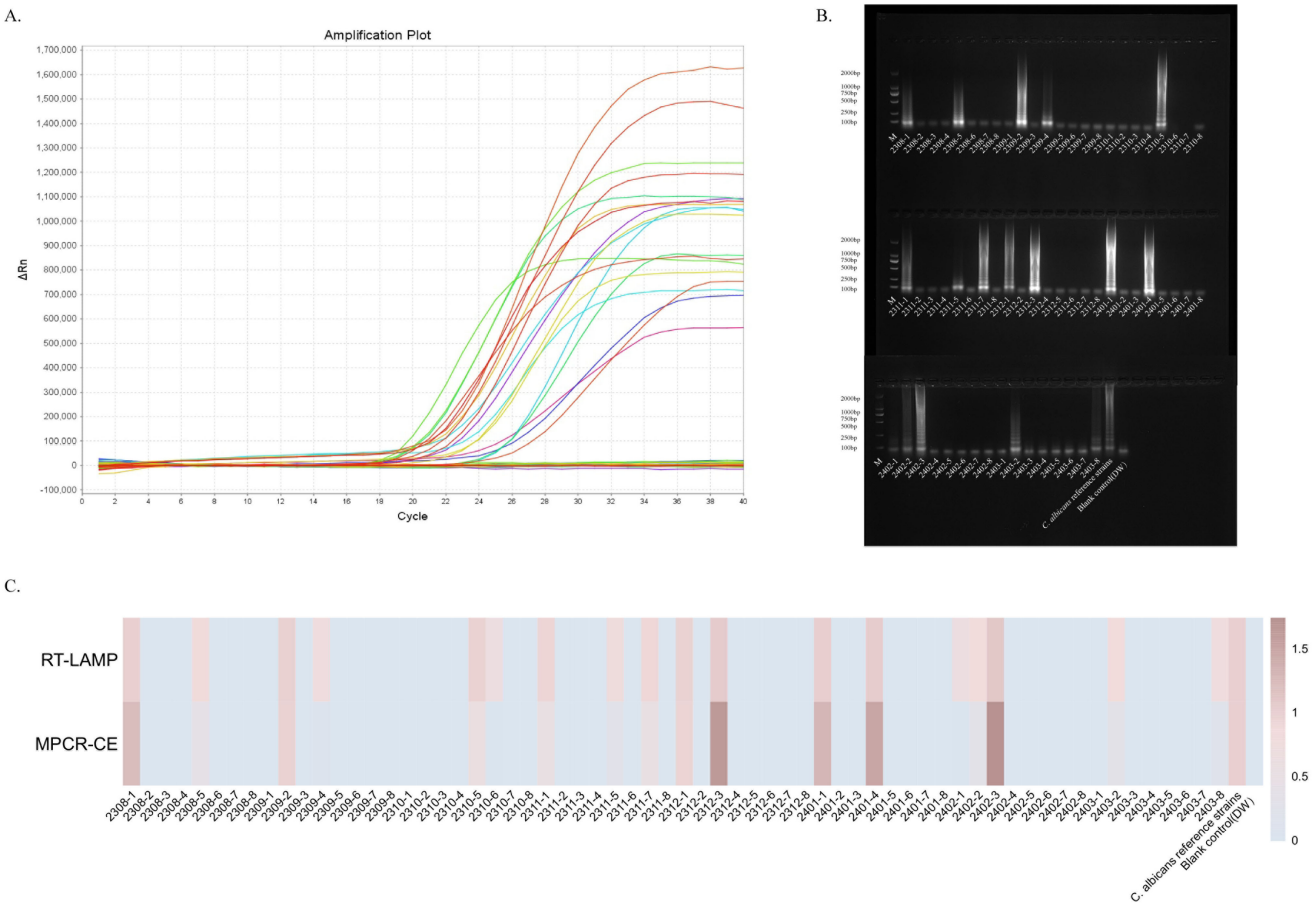
Validation of the ERT-LAMP-CA assay in clinical specimens.ERT-LAMP-CA was used to test 64 clinical specimens from August 2023 to March 2024, with 8 clinical specimens selected each month. The results showed 18 samples were positive **(A)**. Validation of the amplification products using 1.5% agarose gel electrophoresis gave the same results **(B)**. The trend of the relative concentration of *C.albicans* in these 64 clinical samples was consistent with the relative quantification of ERT-LAMP-CA and MPCR-CE **(C)**.

We analysed 64 clinical specimens collected between August 2023 and March 2024 for this technique to provide in-depth validation of the potential application of the ERT-LAMP-CA assay as a *C. albicans* detection tool in clinical diagnostics. **Figure 7A** depicts the corresponding experimental results. Of the 64 clinical samples, 18 samples showed a positive reaction based on the results of the ERT-LAMP-CA assay. The amplification products of these samples were further analysed using agarose gel electrophoresis, and the results are consistent with the fluorescence-LAMP assay in **Figure 7B**. These 18 samples were confirmed positive using culture test verification.

Additionally, these 64 clinical samples were tested for comparative analyses using the MPCR-CE technique, which is routinely used in the current clinical sample (**Supplementary Figure S1**). For data recording, the blank control (DW) results were recorded as 0, and the results of the DNA template of the standard strain were recorded as 1. **Figure 7C** presents the concentration analysis and comparison. The analyses revealed that the ERT-LAMP-CA assay showed similar results as those of the MPCR-CE in concentration detection, and the ERT-LAMP-CA assay demonstrated a higher sensitivity. This study demonstrated that the ERT-LAMP-CA assay is highly sensitive and capable of providing a preliminary estimation of the concentration of *C. albicans* specimens. It also possesses the potential to replace the traditional culture method and the current clinical use of the technique.

## Discussion

In the present context of medical diagnostic practice, the detection of *Candida albicans* is still commonly performed using traditional pathogen culture techniques. It is acknowledged that the identification of pathogens through culture remains the gold standard for diagnosing fungal infectious diseases. However, the traditional cultural method has increasingly become inadequate for meeting the demands of clinical diagnosis. Recently, immunofluorescence (Gunasekera et al., 2015), mass spectrometry (Zehm et al., 2012), and PCR techniques (Kasai et al., 2006) have been employed in clinical settings for the detection of C. albicans. However, these methods require personnel trained in professional instrumentation to operate and analyse the results, which presents a significant challenge to popularisation due to the high requirements for practitioners and laboratory-related instruments.

LAMP technology, a means of isothermal amplification of nucleic acids, has been widely utilised in multiple pathogen detections because of its simplicity and speed (Notomi, 2000). The interpretation of results can be visually assessed by a colour developer that has been pre-positioned in the reaction vessel (Mori et al., 2004) Alternatively, further detection can be facilitated with the assistance of an LFB (Wang et al., 2022). Although these methods are straightforward and rapid, the visual interpretation of colour change is inherently subjective. The subsequent use of biosensors may introduce contamination risk (Huang et al., 2023), and neither of the two mainstream methods provides an estimate of pathogen concentration with any degree of precision. Moreover, the selection of membrane material in the biosensor may influence the test results (Cao et al., 2021).

We developed a new real-time fluorescence-LAMP detection strategy for *C. albicans* that combines the advantages of loop-mediated isothermal amplification and real-time fluorescence detection. The whole reaction process can be monitored in real-time, generating results that take < 1 h, and the reaction system does not need to be opened during the entire amplification process, thus effectively avoiding contamination risk during the detection phase.

Herein, we developed an assay kit of six primers targeting ITS2 sequences for real-time fluorescence-LAMP detection of *C. albicans*. **Figures 3** and **6** depict that the developed primers specifically recognise DNA templates of *C. albicans* strains without cross-reacting with nucleic acids of non-*C. albicans* pathogens or blank control templates. This study verified the detection sensitivity of the ERT-LAMP-CA assay using a serial dilution of *C. albicans* genomic DNA templates. **Figure 5** demonstrates that the lowest detection limit of the ERT-LAMP-CA assay system was approximately 500 ag/μL, which showed higher sensitivity than the pre-laboratory designed LAMP-LFB assay (Wang et al., 2022).

DNA templates from clinical samples were analysed using the ERT-LAMP-CA assay and the MPCR-CE assay, and the assay concentration was readily estimated to assess the potential of the ERT-LAMP-CA assay system in clinical laboratory diagnosis. Of the 64 sputum samples, 18 samples (28.1%) were positive for the ERT-LAMP-CA assay and the MPCR-CE assay, consistent with the results of the traditional culture method, and the concentration estimates of the two methods converged. During the detection process, the ERT-LAMP-CA assay system did not need to open the cap during the amplification process, significantly reducing the contamination risk of amplification products. This problem is difficult to avoid with the LAMP-LFB and MPCR-CE methods.

Additionally, since the results of the ERT-LAMP-CA assay can be reported in real-time using the fluorescent PCR instrument, compared with the LFB assay, which can only distinguish between negative and positive results, the ERT-LAMP-CA assay system can make a preliminary estimation of the specimen concentration, which makes the test results more objective and specific. Therefore, the real-time LAMP assay demonstrated more potential for application in the rapid, sensitive, and specific identification of *C. albicans* infections than the MPCR-CE method currently used in clinical settings and the LAMP-LFB method in pre-laboratory studies.

The agarose electrophoresis of the amplified products revealed that samples with lower concentrations were invisible. However, the ERT-LAMP-CA detection system showed a more pronounced amplification and, thus, a better response to *C. albicans* infection. For example, **Figure 7** indicates that the electrophoresis of specimens 2310-6 amplified products and 2302-1 are inconsistent with those of ERT-LAMP-CA.

This study developed a real-time fluorescence-LAMP assay for *C. albicans* that integrates loop-mediated isothermal amplification technology with real-time fluorescence detection. The method demonstrated several significant advantages when comparing current molecular detection techniques reported in the literature, providing a new solution for rapid, efficient, and reliable clinical detection of *C. albicans* infections.

## Conclusion

The ERT-LAMP-CA assay that targeted the ITS2 gene of *C. albicans* was effectively developed in this study. The ERT-LAMP-CA assay showed reasonable specificity and sensitivity by detecting reference strains and clinical samples in the application and evaluation process. Consequently, the ERT-LAMP-CA assay provides a new option for reliable, rapid, and simple detection of *C. albicans*.

## Data Availability

The original contributions presented in the study are included in the article/**Supplementary Material**. Further inquiries can be directed to the corresponding author/s.
