# Supplementary material for: A novel, rapid, ultrasensitive diagnosis platform for detecting Candida albicans using restriction endonuclease‐mediated real-time loop-mediated isothermal amplification
Source: Front Cell Infect Microbiol. 2024 Nov 11;14:1450199. doi: 10.3389/fcimb.2024.1450199 (PMC11586279; doi:10.3389/fcimb.2024.1450199)
Supplement: Supplementary file 1 [file DataSheet1.docx]

**Supplementary material**

**TABLE S1 | Clinical Sample detail and detection**

| Sample Number | Traditional Cultivation  Method | MPCR-CE  RFU | ERT-LAMP-CA  CT value |
| --- | --- | --- | --- |
| 2308-1 | + | 29.27 | 21.84 |
| 2308-2 | - | 0 | 40 |
| 2308-3 | - | 0 | 40 |
| 2308-4 | - | 0 | 40 |
| 2308-5 | + | 9.73 | 24.02 |
| 2308-6 | - | 0 | 40 |
| 2308-7 | - | 0 | 40 |
| 2308-8 | - | 0 | 40 |
| 2309-1 | - | 0 | 40 |
| 2309-2 | + | 23.17 | 22.2 |
| 2309-3 | - | 0 | 40 |
| 2309-4 | + | 3.07 | 26.45 |
| 2309-5 | - | 0 | 40 |
| 2309-6 | - | 0 | 40 |
| 2309-7 | - | 0 | 40 |
| 2309-8 | - | 0 | 40 |
| 2310-1 | - | 0 | 40 |
| 2310-2 | - | 0 | 40 |
| 2310-3 | - | 0 | 40 |
| 2310-4 | - | 0 | 40 |
| 2310-5 | + | 14.14 | 22.63 |
| 2310-6 | + | 0.5 | 28.04 |
| 2310-7 | - | 0 | 40 |
| 2310-8 | - | 0 | 40 |
| 2311-1 | + | 12.32 | 23.09 |
| 2311-2 | - | 0 | 40 |
| 2311-3 | - | 0 | 40 |
| 2311-4 | - | 0 | 40 |
| 2311-5 | + | 7.29 | 26.35 |
| 2311-6 | - | 0 | 40 |
| 2311-7 | + | 12.23 | 23.46 |
| 2311-8 | - | 0 | 40 |
| 2312-1 | + | 22.81 | 22.41 |
| 2312-2 | - | 0 | 40 |
| 2312-3 | + | 39.42 | 21.06 |
| 2312-4 | - | 0 | 40 |
| 2312-5 | - | 0 | 40 |
| 2312-6 | - | 0 | 40 |
| 2312-7 | - | 0 | 40 |
| Sample Number | Traditional Cultivation  Method | MPCR-CE  RFU | ERT-LAMP-CA  CT value |
| 2312-8 | - | 0 | 40 |
| 2401-1 | + | 31.02 | 21.45 |
| 2401-2 | - | 0 | 40 |
| 2401-3 | - | 0 | 40 |
| 2401-4 | + | 35.35 | 21.16 |
| 2401-5 | - | 0 | 40 |
| 2401-6 | - | 0 | 40 |
| 2401-7 | - | 0 | 40 |
| 2401-8 | - | 0 | 40 |
| 2402-1 | + | 6.53 | 26.04 |
| 2402-2 | + | 12.23 | 24.44 |
| 2402-3 | + | 41.56 | 20.24 |
| 2402-4 | - | 0 | 40 |
| 2402-5 | - | 0 | 40 |
| 2402-6 | - | 0 | 40 |
| 2402-7 | - | 0 | 40 |
| 2402-8 | - | 0 | 40 |
| 2403-1 | - | 0 | 40 |
| 2403-2 | + | 2.52 | 27.13 |
| 2403-3 | - | 0 | 40 |
| 2403-4 | - | 0 | 40 |
| 2403-5 | - | 0 | 40 |
| 2403-6 | - | 0 | 40 |
| 2403-7 | - | 0 | 40 |
| 2403-8 | + | 4.98 | 26.44 |
| C.A | + | 23.79 | 21.94 |
| DW | - | 0 | 40 |

**Abbreviation:**MPCR-CE,multiplex PCR(Polymerase Chain Reaction)-capillary electrophoresis;RFU,Relative Fluorescence Units;ERT-LAMP-CA,*Candida albicans* using restriction endonuclease-mediated real-time loop-mediated isothermal amplification.


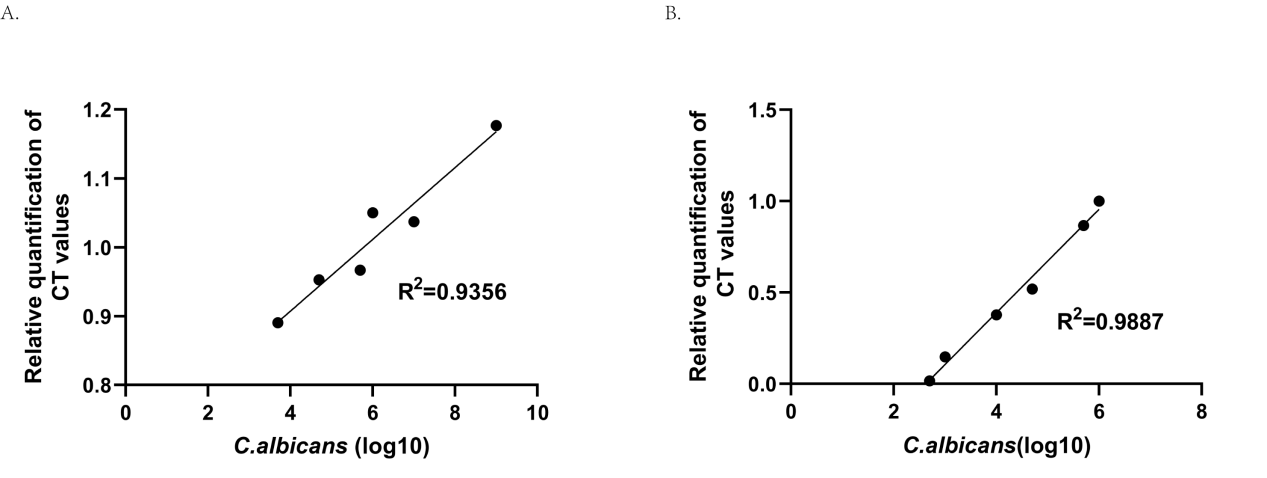


**FIGURE S1 Results of relative linearity analysis of concentration gradients.** To perform this analysis, We plotted the logarithm of the *C. albicans* concentration (LOG_10_) as the X-axis. And the relative quantitative values of the CT values as the Y-axis. By fitting the data linearly, we obtained correlation coefficients (R-values) to assess the strength of the linear relationship. (A) the linear analysis of Genomic DNA was diluted sequentially:1ng/μl,10pg/μl, 1pg/μl,500fg/μl, 50fg/μl, 5fg/μl; (B) the linear analysis of linear analysis of Genomic DNA was diluted sequentially:1pg/μl, 500fg/μl,50fg/μl, 10fg/μl,1fg/μl, 500ag/μl.


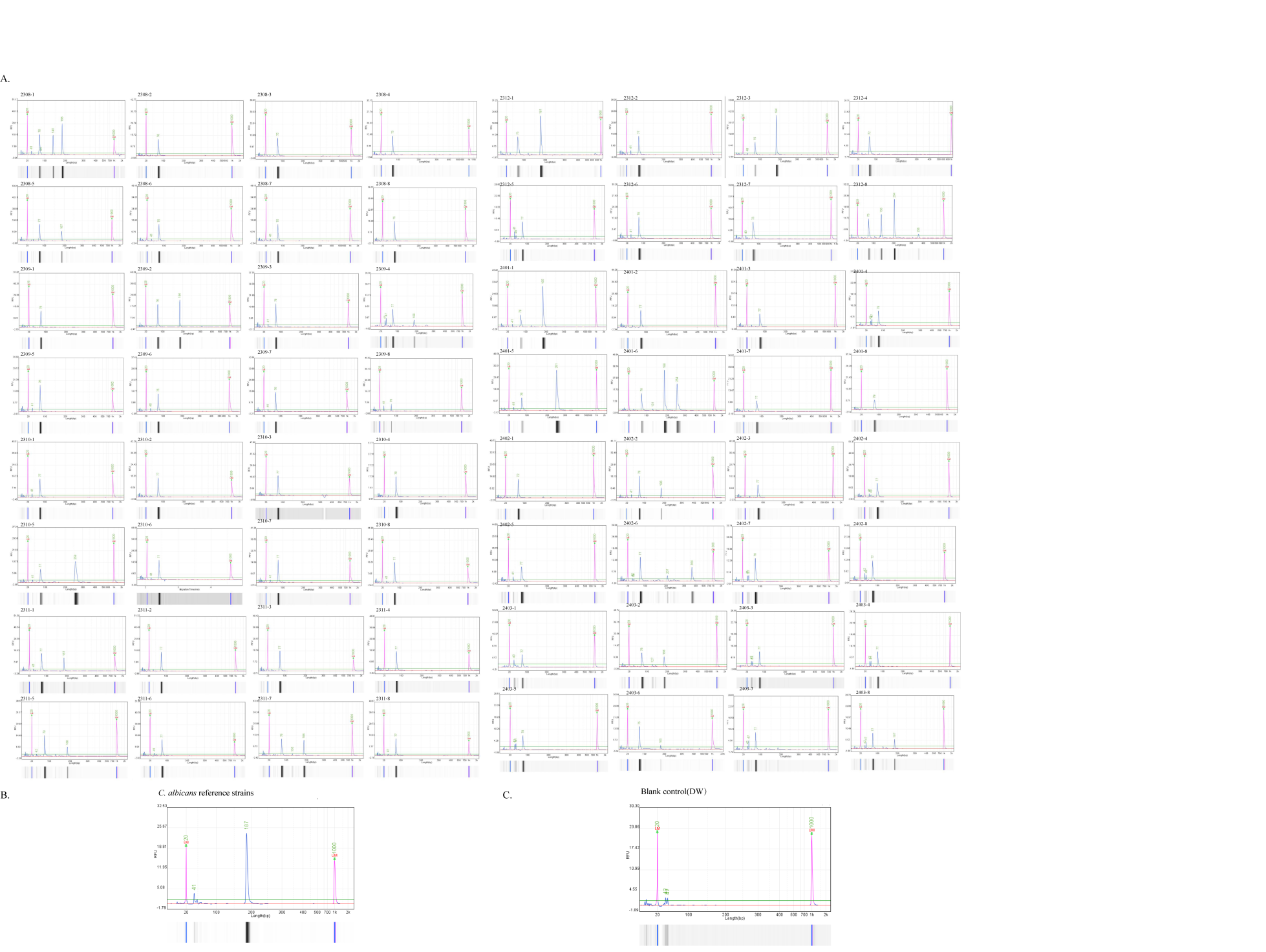


**FIGURE S2 Detection of *C.albicans* using multiplex PCR-capillary electrophoresis.**The sample**s** were subjected to multiplex PCR-capillary electrophoresis after DNA extraction.The 173-193 bp fragment of the reaction product is *C. albicans* specific.(A) Eight clinical samples were selected monthly from August 2023 to March 2024 and DNA was extracted and subjected to multiplex PCR-

capillary electrophoresis.(B) *C.albicans* standard strains. (C)Blank controls (DW).
